# Correction: Modelling locust foraging: How and why food affects group formation

**DOI:** 10.1371/journal.pcbi.1009695

**Published:** 2021-12-20

**Authors:** Fillipe Georgiou, Camille Buhl, J. E. F. Green, Bishnu Lamichhane, Ngamta Thamwattana

There are errors in three equations in S1 Appendix and S3 Appendix. Reference 51 is incomplete. Please view the correct S1 Appendix, S3 Appendix, and Reference 51 below.

**Reference 51**:

Reynolds AM, Sword GA, Simpson SJ, Reynolds DR. Predator percolation, insect outbreaks, and phase polyphenism. Current Biology. 2009;19(1):20-24. pmid:19097898

## Supporting information

S1 Appendix Detailed derivation of local flux. The full detailed derivation of the flux terms based on local interactions given in the model derivation section.
(PDF)

S3 Appendix Numerical scheme. The full detailed derivation of the numerical scheme used for simulating the numerical results.
(PDF)
