# Supplementary material for: Modelling locust foraging: How and why food affects group formation
Source: PLoS Comput Biol. 2021 Jul 7;17(7):e1008353. doi: 10.1371/journal.pcbi.1008353 (PMC8289112; doi:10.1371/journal.pcbi.1008353)
Supplement: S2 Appendix — The full detailed derivations of the analytic results given in the PDE model analysis section. (PDF) [file pcbi.1008353.s002.pdf]

S2 Appendix: Detailed analytic results

Fillipe Georgiou[1*], Camille Buhl[2], J.E.F. Green[3], Bishnu Lamichhane[1], Ngamta Thamwattana[1],

1 School of Mathematical and Physical Sciences, University of Newcastle, Callaghan, Australia
2 School of Agriculture, Food and Wine, University of Adelaide, Adelaide, Australia
3 School of Mathematical Sciences, University of Adelaide, Adelaide, Australia

* fillipe.georgiou@uon.edu.au

In this appendix we provide the full details of the calculations in the PDE model analysis section. To begin our non-dimensionalised system of equations is given by

$$\frac{\partial g}{\partial t} + \nabla \cdot (g\boldsymbol{v}_g) - D\nabla \cdot \left[e^{-c}\nabla g\right] = -f_1(\rho)g + f_2(\rho)s, \tag{1a}$$

$$\frac{\partial s}{\partial t} + \nabla \cdot (s\boldsymbol{v}_s) - D\nabla \cdot \left[De^{-c}\nabla s\right] = f_1(\rho)g - f_2(\rho)s, \tag{1b}$$

$$\frac{\partial c}{\partial t} = -\kappa c(\boldsymbol{x}, t)\rho(\boldsymbol{x}, t), \tag{1c}$$

with

$$\boldsymbol{v}_g = -\nabla(Q_g \star \rho) + De^{-c}\left(\nabla c - \gamma\rho\nabla\rho\right),$$

and

$$\boldsymbol{v}_s = -\nabla(Q_s \star \rho) + De^{-c}\left(\nabla c - \gamma\rho\nabla\rho\right),$$

with our specific functions given by

$$Q_g = R_g e^{\frac{-|\boldsymbol{x}|}{r_g}} - A_g e^{-|\boldsymbol{x}|}, Q_s = R_s e^{\frac{-|\boldsymbol{x}|}{r_s}},$$

$$f_1(\rho) = \frac{\delta^*}{1+\rho^2}, f_2(\rho) = \frac{(\rho k)^2}{1+(\rho k)^2}.$$

We will define the total mass of locusts as

$$M = \int \rho(\boldsymbol{x}, t)\, d\boldsymbol{x}, \tag{2}$$

and the global gregarious mass fraction as

$$\phi_g(t) = \frac{\int g(\boldsymbol{x}, t)\, d\boldsymbol{x}}{M}. \tag{3}$$

We also define the local gregarious mass fraction as

$$\psi_g(\boldsymbol{x}, t) = \frac{g(\boldsymbol{x}, t)}{\rho(\boldsymbol{x}, t)}, \tag{4}$$

It is possible to write s and g in terms of (4) as

$$g = \psi_g(\boldsymbol{x}, t)\rho(\boldsymbol{x}, t) \text{ and } s = (1 - \psi_g(\boldsymbol{x}, t))\rho(\boldsymbol{x}, t).$$

Density of gregarious groups

By making a few simplifying assumptions we can estimate the maximum density and width of gregarious locusts in both the large and small mass limits in one dimension. To begin, our assumptions are c is constant and not depleting, there are minimal solitarious locusts present in the group (i.e. $\rho \approx g$), and the effect of phase transitions in the group is negligible (i.e. $f_1(\rho)s = f_2(\rho)g = 0$). Finally, we will label the support of g as Ω'. These assumptions give,

$$\frac{\partial g}{\partial t} + \nabla \cdot (g\boldsymbol{v}_g) = 0,$$

with

$$\boldsymbol{v}_g = -\nabla(Q_g \star g) - De^{-c}\gamma g \nabla g - De^{-c}\nabla \log(g).$$

We can then rewrite $\boldsymbol{v}_g$ as,

$$\boldsymbol{v}_g = -\nabla \left[(Q_g \star g) + \frac{De^{-c}\gamma}{2} g^2 + De^{-c} \log(g) \right].$$

Which is a gradient flow of the form

$$\frac{\partial g}{\partial t} = \nabla \cdot \left(g \nabla \left[\frac{\delta E}{\delta g} \right] \right),$$

where

$$E[g] = \int_{\Omega'} \frac{1}{2} g [Q_g \star g] + \frac{De^{-c}\gamma}{6} g^3 + De^{-c}(g \log(g) - g)\, dx, \tag{5}$$

with the minimisers satisfying

$$\frac{\delta E}{\delta g} = (Q_g \star g) + \frac{De^{-c}\gamma}{2} g^2 + De^{-c} \log(g) = \lambda.$$

Next, we will follow the work of [1, 3, 4] and with a series of simplifying assumptions we consider the large mass limit and small mass limit in turn. For the two limits, while the support of g, Ω', is infinite due to the linear diffusion the bulk of the mass is contained as a series of aggregations, we will approximate the support of an aggregation as Ω. We also note that (2) becomes

$$M = \int_{\Omega} \rho(x)\, dx = \int_{\Omega} g(x)\, dx.$$

Large Mass Limit

Beginning with (5) we assume the following; $g(x)$ is approximately rectangular, and for a single aggregation we assume that the support is far larger than the range of attraction of $e^{\frac{-|x|}{r}}$. We thus make the approximation, $e^{\frac{-|x|}{r}} \approx 2r\delta(x)$ (where $\delta(x)$ is the Dirac delta function) to ensure that the volume of the integration is preserved, and therefore $Q_g \approx 2\left(R_g r_g - A_g\right)\delta(x)$. Next, as g is rectangular

$$||\Omega|| = \frac{M}{g}.$$

Substituting into (5) we get

$$E[g] = M \left(\left(R_g r_g - A_g\right) g + \frac{De^{-c}\gamma}{6} g^2 + De^{-c}(\log(g) - 1) \right).$$

We can then find

$$\frac{dE}{dg} = M\left((R_g r_g - A_g) + \frac{De^{-c}\gamma}{3} g + \frac{De^{-c}}{g}\right),$$

which has critical point at

$$\frac{De^{-c}\gamma}{3} g^2 + (R_g r_g - A_g)\, g + De^{-c} = 0.$$

Thus

$$g = \frac{3\left(-\left(R_g r_g - A_g\right) \pm \sqrt{\left(R_g r_g - A_g\right)^2 - \frac{4(De^{-c})^2\gamma}{3}}\right)}{2De^{-c}\gamma}.$$

Based on numerical simulations we take only the positive root, then as the solution is constant

$$||g||_\infty = \frac{3\left(-\left(R_g r_g - A_g\right) + \sqrt{\left(R_g r_g - A_g\right)^2 - \frac{4(De^{-c})^2\gamma}{3}}\right)}{2De^{-c}\gamma}, \quad (6)$$

with support

$$||\Omega|| = \frac{2MDe^{-c}\gamma}{3\left(-\left(R_g r_g - A_g\right) + \sqrt{\left(R_g r_g - A_g\right)^2 - \frac{4(De^{-c})^2\gamma}{3}}\right)}. \quad (7)$$

Small Mass Limit

Beginning with (5) and using the following simplifying assumptions; For a single aggregation we can approximate the social interaction potential using a Taylor expansion, $e^{\frac{-|x|}{r}} \approx 1 - \frac{|x|}{r}$, and therefore $Q_g \approx (R_g - A_g) - |x|\left(\frac{R_g}{r_g} - A_g\right)$. Additionally, we will ignore the effect of linear diffusion within Ω, giving (5) as

$$E[g] = \int_\Omega \frac{1}{2} g[Q_g \star g] + \frac{De^{-c}\gamma}{6} g^3\, dx. \quad (8)$$

Based on these assumptions we can find

$$\frac{\delta E}{\delta g} = \left((R_g - A_g) - |x|\left(\frac{R_g}{r_g} - A_g\right)\right) \star g + \frac{De^{-c}\gamma}{2} g^2 = \lambda,$$

which becomes

$$(R_g - A_g)M - \left(\frac{R_g}{r_g} - A_g\right)(|x| \star g) + \frac{De^{-c}\gamma}{2}g^2 = \lambda.$$

We then exploit the property that $(|x|)_{xx} = 2\delta(x)$ and differentiate twice to obtain

$$-2\left(\frac{R_g}{r_g} - A_g\right)g + \frac{De^{-c}\gamma}{2}(g^2)_{xx} = 0.$$

Following [3] we place the maximum of g at the origin; this implies $g_x(0) = 0$ and $g(0) = ||g||_\infty$. We then let,

$$p = \frac{g}{||g||_\infty}, \text{ and } \zeta = \frac{x}{\sqrt{||g||_\infty}}, \tag{9}$$

giving,

$$(p^2)_{\zeta\zeta} - \frac{4\left(\frac{R_g}{r_g} - A_g\right)}{De^{-c}\gamma}p = 0, \ p(0) = 1, \ p_\zeta(0) = 0.$$

We then multiply through by $(p^2)_\zeta$ and integrate to obtain,

$$2p^2(p)_\zeta^2 - \frac{8\left(\frac{R_g}{r_g} - A_g\right)}{3De^{-c}\gamma}p^3 + c = 0.$$

Then applying the conditions at $\zeta = 0$ we find,

$$2p^2(p)_\zeta^2 - \frac{8\left(\frac{R_g}{r_g} - A_g\right)}{3De^{-c}\gamma}(p^3 - 1) = 0,$$

which can be simplified into,

$$p_\zeta = \sqrt{\frac{4\left(A_g - \frac{R_g}{r_g}\right)}{3De^{-c}\gamma}\left(\frac{1}{p^2} - p\right)}.$$

Performing a separation of variables gives

$$d\zeta = \sqrt{\frac{3De^{-c}\gamma}{4\left(A_g - \frac{R_g}{r_g}\right)}}\frac{p\,dp}{\sqrt{1 - p^3}} \tag{10}$$

We can then find the implicit solution,

$$\zeta = \sqrt{\frac{3De^{-c}\gamma}{4\left(A_g - \frac{R_g}{r_g}\right)}} \int_p^1 \frac{p\,dp}{\sqrt{1-p^3}}.$$

As $p \to 0$, $\zeta \to \frac{||\Omega||}{2\sqrt{||g||_\infty}}$, giving

$$\begin{aligned} ||\Omega|| &= 2\sqrt{||g||_\infty}\sqrt{\frac{3De^{-c}\gamma}{4\left(A_g - \frac{R_g}{r_g}\right)}} \int_0^1 \frac{p\,dp}{\sqrt{1-p^3}}, \\ &= 2\sqrt{||g||_\infty}\sqrt{\frac{3De^{-c}\gamma}{4\left(A_g - \frac{R_g}{r_g}\right)}}\frac{1}{3}B\left(\frac{2}{3},\frac{1}{2}\right), \\ &= \sqrt{||g||_\infty \frac{De^{-c}\gamma}{3\left(A_g - \frac{R_g}{r_g}\right)}}B\left(\frac{2}{3},\frac{1}{2}\right), \end{aligned} \tag{11}$$

where B is the β-function (for definition see [5], page 207). Next using the mass constraint,

$$M = 2\int_0^{\frac{||\Omega||}{2}} g(x)\,dx = 2\int_{-\frac{||\Omega||}{2}}^0 g(x)\,dx,$$

and substituting (9) we obtain

$$M = 2||g||_\infty^{\frac{3}{2}} \int_{-\frac{||\Omega||}{2\sqrt{||g||_\infty}}}^0 p(\zeta)\,d\zeta,$$

which using (10) becomes,

$$\begin{aligned} M &= ||g||_\infty^{\frac{3}{2}}\sqrt{\frac{3De^{-c}\gamma}{\left(A_g - \frac{R_g}{r_g}\right)}} \int_0^1 \frac{p^2\,dp}{\sqrt{1-p^3}}, \\ &= ||g||_\infty^{\frac{3}{2}}\sqrt{\frac{4De^{-c}\gamma}{3\left(A_g - \frac{R_g}{r_g}\right)}}. \end{aligned} \tag{12}$$

Then using (11) and (12) we can find $||\Omega||$ and $||g||_\infty$ in terms of M, giving

$$||g||_\infty = \sqrt[3]{\frac{3M^2\left(A_g - \frac{R_g}{r_g}\right)}{4De^{-c}\gamma}}, \tag{13}$$

and

$$||\Omega|| = B\left(\frac{2}{3}, \frac{1}{2}\right) \sqrt[3]{\frac{MDe^{-c}\gamma}{6\left(A_g - \frac{R_g}{r_g}\right)}}. \tag{14}$$

Linear stability analysis of homogeneous steady states

In order to gain insights into the conditions under which groups can form, we investigate the stability of spatially-homogeneous steady states. In this analysis we perturb the homogeneous steady states by adding a small amount of noise. We then find under what conditions the small perturbations grow and are likely to lead to groups. We begin by defining the homogeneous steady states of s, g, and c, as $\bar{s}$, $\bar{g}$, and $\bar{c}$, with the total density given as $\bar{\rho} = \bar{s} + \bar{g}$. We again assume that c does not deplete (i.e. $\kappa = 0$). As we are assuming either an infinite or periodic domain, we must redefine the global gregarious mass fraction, (3), as

$$\phi_g(t) = \frac{g(t)}{\rho(t)}. \tag{15}$$

. Let

$$s = \bar{s} + \epsilon\tilde{s},\ g = \bar{g} + \epsilon\tilde{g},\ \text{and}\ c = \bar{c} + \epsilon\tilde{c},$$

where $\epsilon \ll 1$, $\bar{\cdot}$ represents a homogeneous steady state and $\epsilon\tilde{\cdot}$ represents a small perturbation. Naturally,

$$\rho = \bar{\rho} + \epsilon\tilde{\rho} = \bar{s} + \bar{g} + \epsilon(\tilde{s} + \tilde{g}).$$

Substituting this into (1a), (1b), and (1c), performing a Taylor expansion on $f_1(\bar{\rho} + \epsilon\tilde{\rho})$, $f_2(\bar{\rho} + \epsilon\tilde{\rho})$ and $De^{-(\bar{c}+\epsilon\tilde{c})}$, neglecting terms of $O(\epsilon^2)$ and higher, and for notational convenience letting $\hat{D} = De^{-\bar{c}}$, gives

$$\frac{\partial}{\partial t}\begin{bmatrix}\tilde{s}\\ \tilde{g}\\ \tilde{c}\end{bmatrix} = \begin{bmatrix}-\bar{s}\left[-Q_s * \nabla^2(\tilde{s}+\tilde{g}) + \hat{D}\left(\nabla^2\tilde{c} - \gamma\bar{\rho}\nabla^2(\tilde{s}+\tilde{g})\right)\right] + \hat{D}\Delta(\tilde{s}) - \tilde{s}A + \tilde{g}B\\ -\bar{g}\left[-Q_g * \nabla^2(\tilde{s}+\tilde{g}) + \hat{D}\left(\nabla^2\tilde{c} - \gamma\bar{\rho}\nabla^2(\tilde{s}+\tilde{g})\right)\right] + \hat{D}\Delta(\tilde{g}) + \tilde{s}A - \tilde{g}B\\ 0\end{bmatrix},$$

where

$$A = f_2(\bar{\rho}) + f_2'(\bar{\rho})\bar{s} - f_1'(\bar{\rho})\bar{g},$$
$$B = f_1(\bar{\rho}) - f_2'(\bar{\rho})\bar{s} + f_1'(\bar{\rho})\bar{g}.$$

We then perform a Fourier series expansion of $\tilde{s}$, $\tilde{g}$, and $\tilde{c}$,

$$\tilde{s} = \sum_{\hat{k}} S_{\hat{k}}(t)e^{i\hat{k}x},\ \tilde{g} = \sum_{\hat{k}} G_{\hat{k}}(t)e^{i\hat{k}x},\ \text{and } \tilde{c} = \sum_{\hat{k}} C_{\hat{k}}(t)e^{i\hat{k}x},$$

as well as taking the Fourier transform of Q_s and Q_g denoted as $\hat{Q}_s$ and $\hat{Q}_g$. This gives,

$$\frac{\partial}{\partial t}\begin{bmatrix} S_{\hat{k}} \\ G_{\hat{k}} \\ C_{\hat{k}} \end{bmatrix} = \begin{bmatrix} -\bar{s}\hat{k}^2\left(\hat{Q}_s + \gamma\hat{D}\right) - \hat{k}^2\hat{D} - A & -\bar{s}\hat{k}^2\left(\hat{Q}_s + \gamma\bar{\rho}\hat{D}\right) + B & \hat{D}\bar{s}\hat{k}^2 \\ -\bar{g}\hat{k}^2\left(\hat{Q}_g + \gamma\hat{D}\right) + A & -\bar{g}\hat{k}^2\left(\hat{Q}_g + \gamma\bar{\rho}\hat{D}\right) - \hat{k}^2\hat{D} - B & \hat{D}\bar{g}\hat{k}^2 \\ 0 & 0 & 0 \end{bmatrix}\begin{bmatrix} S_{\hat{k}} \\ G_{\hat{k}} \\ C_{\hat{k}} \end{bmatrix}.$$

We find the eigenvalues of the coefficient matrix as

$$\lambda_1 = -\hat{D}\hat{k}^2 - f_1(\bar{\rho}) - f_2(\bar{\rho}),\ \lambda_2 = -\hat{D}\hat{k}^2 - \bar{g}\hat{k}^2(\hat{D}\bar{\rho}\gamma + \hat{Q}_g) - \bar{s}\hat{k}^2(\hat{D}\bar{\rho}\gamma + \hat{Q}_s),\ \text{and } \lambda_3 = 0.$$

To determine the conditions under which the homogeneous steady state is unstable to small perturbations and thus likely to lead to aggregations, we need to find a $\hat{k}$ such that λ_1, λ_2 or λ_3 is greater than 0. As $f_1(\rho), f_2(\rho)$ are positive functions, $\lambda_1 < 0 \,\forall\, \hat{k}$ and $\lambda_3 = 0$. For λ_2, we need,

$$-\hat{D}\hat{k}^2 - \bar{g}\hat{k}^2(\hat{D}\bar{\rho}\gamma + \hat{Q}_g) - \bar{s}\hat{k}^2(\hat{D}\bar{\rho}\gamma + \hat{Q}_s) > 0,$$
$$-\bar{g}(\hat{D}\bar{\rho}\gamma + \hat{Q}_g) - \bar{s}(\hat{D}\bar{\rho}\gamma + \hat{Q}_s) > \hat{D}.$$

Then by rewriting $\bar{s}$ and $\bar{g}$ in terms of the global gregarious mass fraction (15) and the total density $\bar{\rho}$ [2],

$$\bar{g} = \phi_g\bar{\rho},\ \text{and } \bar{s} = (1 - \phi_g)\bar{\rho}.$$

This gives

$$-\phi_g\bar{\rho}(\hat{D}\bar{\rho}\gamma + \hat{Q}_g) - (1 - \phi_g)\bar{\rho}(\hat{D}\bar{\rho}\gamma + \hat{Q}_s) > \hat{D},$$

where by taking $-\bar{\rho}$ as a common factor gives,

$$-\bar{\rho}\left[\phi_g(\hat{D}\bar{\rho}\gamma+\hat{Q}_g)+(1-\phi_g)(\hat{D}\bar{\rho}\gamma+\hat{Q}_s)\right]>\hat{D}.$$

Next, we re-arrange to make ϕ_g the object of the inequality to give

$$\phi_g>\bar{\phi}_g=\frac{\frac{\hat{D}}{\bar{\rho}}+\hat{D}\bar{\rho}\gamma+\hat{Q}_s}{\hat{Q}_s-\hat{Q}_g}. \tag{16}$$

From this we can see that as the amount of available food increases the gregarious fraction required for group formation decreases. In addition as $\bar{\rho}$ increases the gregarious fraction required for group formation increasing suggesting an upper locust density in order to transition away from the homogeneous steady state.

For our specific functions $Q_g = R_g e^{-\frac{|x|}{r_g}} - A_g e^{-|x|}$ and $Q_s = R_s e^{-\frac{|x|}{r_s}}$, we begin by taking the one dimensional Fourier transforms of Q_s and Q_g using the following definition,

$$\hat{f}(\hat{k})=\int_{\mathbb{R}^n} f(\boldsymbol{x})e^{-i\hat{k}\cdot\boldsymbol{x}}\,d\boldsymbol{x},$$

to get

$$\hat{Q}_g=\frac{2R_g r_g}{1+r_g^2\hat{k}^2}-\frac{2A_g}{1+\hat{k}^2},\quad \hat{Q}_s=\frac{2R_s r_s}{1+r_s^2\hat{k}^2}.$$

As Q_s and $-Q_g$ have a maximum value at $\hat{k}=0$, we let $\hat{k}=0$ and substitute into (16), which gives,

$$\phi_g>\bar{\phi}_g=\frac{\frac{\hat{D}}{\bar{\rho}}+\hat{D}\bar{\rho}\gamma+2R_s r_s}{2A_g-2R_g r_g+2R_s r_s}. \tag{17}$$

From this we can find the maximum homogenous density, $\bar{\rho}$, that locust aggregations can still form. So taking (17) and substituting $\phi_g=1$ gives,

$$1=\frac{\frac{\hat{D}}{\bar{\rho}}+\hat{D}\bar{\rho}\gamma+2R_s r_s}{2A_g-2R_g r_g+2R_s r_s}.$$

Which gives

$$\hat{D}\gamma\bar{\rho}^2-2(A_g-R_g r_g)\bar{\rho}+\hat{D}=0,$$

and this has solutions

$$\bar{\rho} = \frac{(A_g - R_g r_g) \pm \sqrt{(A_g - R_g r_g)^2 - (\hat{D})^2\gamma}}{\hat{D}\gamma} \approx \frac{2}{3}||g||_\infty,$$

where $||g||_\infty$ is given by (6).

We also calculate if it is possible for a particular homogenous density of locusts to form a group by first calculating the homogeneous steady states of s and g using,

$$\frac{\partial g}{\partial t} = -f_1(\bar{\rho})g + f_2(\bar{\rho})s. \tag{18}$$

Then, rewriting s and$g$ in terms of the gregarious mass fraction and solving for the steady state, we find

$$\phi_g = \frac{f_2(\bar{\rho})}{f_1(\bar{\rho}) + f_2(\bar{\rho})}.$$

By combining () with (17) we obtain an implicit condition for group formation as

$$\frac{f_2(\bar{\rho})}{f_1(\bar{\rho}) + f_2(\bar{\rho})} > \frac{\frac{De^{-\bar{c}}}{\bar{\rho}} + De^{-\bar{c}}\bar{\rho}\gamma + 2R_s r_s}{2A_g - 2R_g r_g + 2R_s r_s}. \tag{19}$$

In (19), if the values on the left are not greater than those on the right then it is not possible for a great enough fraction of locusts to become gregarious and for instabilities to occur (and thus form a group). As the right hand sides dependency on locust density decreases as the amount of food increases (the $De^{-\bar{c}}\bar{\rho}\gamma$ term), we can deduce that the presence of food lowers the required density for group formation.

Time dependent properties of homogeneous densities

We also estimate time until group formation with homogeneous locust densities and a constant c. By assuming that s and g are homogeneous we can ignore the spatial components of (1a) and (1b). We again denote the combined homogeneous locust density as $\bar{\rho}$ however now $\bar{\rho} = s(t) + g(t)$. Finally, assuming that $g(0) = 0$, we find the homogeneous density of gregarious locusts as a function of time is given by

$$g(t) = \frac{\bar{\rho} f_2(\bar{\rho})}{f_1(\bar{\rho}) + f_2(\bar{\rho})}\left(1 - e^{-[f_1(\bar{\rho}) + f_2(\bar{\rho})]t}\right).$$

Which we then solve for t^* such that $g(t^*) = \hat{\phi_g}\bar{\rho}$, where $\hat{\phi_g}$ is given by (17). This gives an estimation for time of group formation as,

$$t^* = \frac{-\ln\left(1 - \frac{\hat{\phi_g}(f_1(\bar{\rho})+f_2(\bar{\rho}))}{f_2(\bar{\rho})}\right)}{f_1(\bar{\rho}) + f_2(\bar{\rho})}. \tag{20}$$

Thus, as increasing food decreases the gregarious mass fraction required for group formation it follows that it also decreases the time required for group formation.

Center of mass

Another property of the model to be investigated is how the center of mass for the locusts behaves. For a single population with diffusive terms it has been shown that the center of mass is conserved [1]. Here, we consider how the total population of locusts behaves with a constant food source, i.e. $c(\boldsymbol{x}, t)$ is constant in space and time. We assume that our domain is $\Omega' = \mathbb{R}^n$ with $\rho(\boldsymbol{x}, t) \to 0$ at infinity and a bounded mass M. Finally, Q_s and Q_g are symmetric. To begin, we add (1a) and (1b), and let $\hat{D} = De^{-c}$, to obtain,

$$\frac{\partial(g+s)}{\partial t} + \nabla \cdot (g\boldsymbol{v}_g + s\boldsymbol{v}_s) - \hat{D}\nabla \cdot [\nabla(g+s)] = 0.$$

where

$$\boldsymbol{v}_g = -\nabla(Q_g \star \rho) + \hat{D}\left(\nabla c - \gamma\rho\nabla\rho\right),$$

and

$$\boldsymbol{v}_s = -\nabla(Q_s \star \rho) + \hat{D}\left(\nabla c - \gamma\rho\nabla\rho\right).$$

Then rewriting the equations in terms of the local gregarious mass fraction (4), we obtain

$$\frac{\partial \rho}{\partial t} + \nabla \cdot ((\boldsymbol{v}_g(1-\psi_g) + \boldsymbol{v}_s\psi_g)\rho) - \hat{D}\nabla \cdot [\nabla\rho] = 0.$$

Next, we expand $\boldsymbol{v}_g$ and $\boldsymbol{v}_s$ to get,

$$\frac{\partial \rho}{\partial t} = -\nabla \cdot \left[-\nabla(Q_s \star \rho)\rho + \nabla(Q_s \star \rho)\psi_g\rho - \nabla(Q_g \star \rho)\psi_g\rho - \gamma\hat{D}\rho^2\nabla\rho - \hat{D}\nabla\rho\right].$$

We now look at the behaviour of the center of mass. For notational simplicity we let

$$\langle a, b\rangle = \int_{\Omega'} ab\, d\boldsymbol{x}.$$

Then, (2) can be written as,

$$M = \langle \rho, 1\rangle,$$

and the center of mass, C, of ρ, can be found as

$$C = \frac{1}{M}\langle \rho, \boldsymbol{x}\rangle.$$

To see if the center of mass is conserved, we evaluate

$$\begin{aligned}
M\frac{\partial C}{\partial t} &= \left\langle \frac{\partial \rho}{\partial t}, \boldsymbol{x} \right\rangle, \\
&= \Big\langle -\nabla \cdot \big[-\nabla(Q_s \star \rho)\rho + \nabla(Q_s \star \rho)\psi_g \rho - \nabla(Q_g \star \rho)\psi_g \rho \\
&\quad -\gamma \hat{D}\rho^2 \nabla\rho - \hat{D}\nabla\rho \big], \boldsymbol{x} \Big\rangle, \\
&= \Big\langle -\nabla(Q_s \star \rho)\rho + \nabla(Q_s \star \rho)\psi_g \rho - \nabla(Q_g \star \rho)\psi_g \rho \\
&\quad -\gamma \hat{D}\rho^2 \nabla\rho - \hat{D}\nabla\rho, 1 \Big\rangle, \\
&= \langle -\nabla(Q_s \star \rho), \rho\rangle + \langle \nabla(Q_s \star \rho), \psi_g \rho\rangle - \langle \nabla(Q_g \star \rho), \psi_g \rho\rangle \\
&\quad - \left\langle \gamma \hat{D}\rho^2 \nabla\rho, 1 \right\rangle - \left\langle \hat{D}\nabla\rho, 1 \right\rangle.
\end{aligned}$$

Starting with the diffusion terms, we get

$$\begin{aligned}
-\left\langle \hat{D}\nabla\rho, 1\right\rangle - \left\langle \gamma\hat{D}\rho^2\nabla\rho, 1\right\rangle &= -\left\langle \hat{D}\nabla\rho, 1\right\rangle - \left\langle \frac{\gamma\hat{D}}{3}\nabla\rho^3, 1\right\rangle, \\
&= -\left\langle \frac{\gamma\hat{D}}{3}\rho^3, 0\right\rangle - \left\langle \hat{D}\rho, 0\right\rangle, \\
&= 0.
\end{aligned}$$

This gives

$$M\frac{\partial C}{\partial t} = \langle -\nabla(Q_s \star \rho), \rho\rangle + \langle \nabla(Q_s \star \rho), \psi_g \rho\rangle - \langle \nabla(Q_g \star \rho), \psi_g \rho\rangle.$$

Then using integration by parts we find

$$M\frac{\partial C}{\partial t} = \langle Q_s \star \rho, \nabla(\rho)\rangle - \langle Q_s \star \rho, \nabla(\psi_g \rho)\rangle + \langle Q_g \star \rho, \nabla(\psi_g \rho)\rangle \,. \tag{21}$$

However, using properties of convolutions, specifically $\nabla(Q_s \star \rho) = \nabla(Q_s) \star \rho = Q_s \star \nabla(\rho)$ and the assumption Q_s and Q_g are symmetric, we find

$$\begin{aligned} M\frac{\partial C}{\partial t} &= -\langle \nabla(Q_s \star \rho), \rho\rangle + \langle \nabla(Q_s \star \rho), \psi_g \rho\rangle - \langle \nabla(Q_g \star \rho), \psi_g \rho\rangle \,, \\ &= -\langle (\nabla Q_s) \star \rho), \rho\rangle + \langle (\nabla Q_s) \star \rho, \psi_g \rho\rangle - \langle (\nabla Q_g) \star \rho, \psi_g \rho\rangle \,, \\ &= -\langle \rho, (\nabla Q_s) \star \rho\rangle + \langle \rho, (\nabla Q_s) \star \psi_g \rho\rangle - \langle \rho, (\nabla Q_g) \star \psi_g \rho\rangle \,, \\ &= -\langle \rho, Q_s \star \nabla(\rho)\rangle + \langle \rho, Q_s \star \nabla(\psi_g \rho)\rangle - \langle \rho, Q_g \star \nabla(\psi_g \rho)\rangle \,, \\ &= -\langle Q_s \star \rho, \nabla(\rho)\rangle + \langle Q_s \star \rho, \nabla(\psi_g \rho)\rangle - \langle Q_g \star \rho, \nabla(\psi_g \rho)\rangle \,. \end{aligned} \tag{22}$$

Summing (21) and (22) we get

$$\begin{aligned} 2M\frac{\partial C}{\partial t} &= \langle Q_s \star \rho, \nabla(\rho)\rangle - \langle Q_s \star \rho, \nabla(\psi_g \rho)\rangle + \langle Q_g \star \rho, \nabla(\psi_g \rho)\rangle \\ &\quad - \langle Q_s \star \rho, \nabla(\rho)\rangle + \langle Q_s \star \rho, \nabla(\psi_g \rho)\rangle - \langle Q_g \star \rho, \nabla(\psi_g \rho)\rangle \,. \end{aligned}$$

Thus,

$$\frac{\partial C}{\partial t} = 0.$$

From this we can conclude that in absence of other movement mechanisms (such as alignment) the center of mass of locusts would only move due to food sources.

References

1. Chad M. Topaz, Andrea L. Bertozzi, and Mark A. Lewis. A nonlocal continuum model for biological aggregation. *Bulletin of Mathematical Biology*, 68(7):1601, Jul 2006.

2. Chad M. Topaz, Maria R. D'Orsogna, Leah Edelstein-Keshet, and Andrew J. Bernoff. Locust dynamics: Behavioral phase change and swarming. *PLOS*

Computational Biology, 8(8):e1002642, Aug 2012.

3. Andrew J. Bernoff and Chad M. Topaz. Biological aggregation driven by social and environmental factors: A nonlocal model and its degenerate cahn–hilliard approximation. *SIAM Journal on Applied Dynamical Systems*, 15(3):1528–1562, Jan 2016.

4. Martin Burger, Razvan Fetecau, and Yanghong Huang. Stationary states and asymptotic behavior of aggregation models with nonlinear local repulsion. *SIAM Journal on Applied Dynamical Systems*, 13(1):397–424, Jan 2014.

5. Eric W. Weisstein. *CRC Concise Encyclopedia of Mathematics*. CRC Press, Dec 2002.
