# Supplementary material for: Modelling locust foraging: How and why food affects group formation
Source: PLoS Comput Biol. 2021 Jul 7;17(7):e1008353. doi: 10.1371/journal.pcbi.1008353 (PMC8289112; doi:10.1371/journal.pcbi.1008353)
Supplement: S4 Appendix — Derivation of instantaneous relative advantage from the marginal value theorem. (PDF) [file pcbi.1008353.s004.pdf]

S4 Appendix: Foraging Efficiency

Fillipe Georgiou[1*], Camille Buhl[2], J.E.F. Green[3], Bishnu Lamichhane[1], Ngamta Thamwattana[1],

1 School of Mathematical and Physical Sciences, University of Newcastle, Callaghan, Australia
2 School of Agriculture, Food and Wine, University of Adelaide, Adelaide, Australia
3 School of Mathematical Sciences, University of Adelaide, Adelaide, Australia

* fillipe.georgiou@uon.edu.au

In this appendix we provide a more detailed argument of the foraging efficiency measure used. First, recall that our non-dimensionalised system of equations are given by

$$\frac{\partial g}{\partial t} + \nabla \cdot (g\boldsymbol{v}_g) - D\nabla \cdot \left[e^{-c}\nabla g\right] = -f_1(\rho)g + f_2(\rho)s, \tag{1a}$$

$$\frac{\partial s}{\partial t} + \nabla \cdot (s\boldsymbol{v}_s) - D\nabla \cdot \left[De^{-c}\nabla s\right] = f_1(\rho)g - f_2(\rho)s, \tag{1b}$$

$$\frac{\partial c}{\partial t} = -\kappa c(\boldsymbol{x},t)\rho(\boldsymbol{x},t), \tag{1c}$$

where

$$\boldsymbol{v}_g = -\nabla(Q_g \star \rho) + De^{-c}\left(\nabla c - \gamma\rho\nabla\rho\right),$$

and

$$\boldsymbol{v}_s = -\nabla(Q_s \star \rho) + De^{-c}\left(\nabla c - \gamma\rho\nabla\rho\right),$$

with our specific functions given by

$$Q_g = R_g e^{\frac{-|\boldsymbol{x}|}{r_g}} - A_g e^{-|\boldsymbol{x}|},\ Q_s = R_s e^{\frac{-|\boldsymbol{x}|}{r_s}},$$

$$f_1(\rho) = \frac{\delta^*}{1+\rho^2},\ f_2(\rho) = \frac{(\rho k)^2}{1+(\rho k)^2}.$$

We also define the total mass of locusts as

$$M = \int \rho(\boldsymbol{x}, t)\, d\boldsymbol{x} \tag{2}$$

and the global gregarious mass fraction as

$$\phi_g(t) = \frac{\int g(\boldsymbol{x}, t)\, d\boldsymbol{x}}{M}. \tag{3}$$

Finally, we give the measures of foraging efficiency defined in [1]. The per capita contact with food for solitarious and gregarious locusts, respectively are given by

$$\eta_s(t) = \frac{1}{M} \int_0^L \frac{c(x,t)s(x,t)}{(1-\phi_g(t))} dx \text{ and } \eta_g(t) = \frac{1}{M} \int_0^L \frac{c(x,t)g(x,t)}{\phi_g(t)} dx,$$

where M is given by (2). The instantaneous relative advantage at time t is given by

$$b(t) = \frac{\eta_g(t)}{\eta_s(t)}. \tag{4}$$

1 Foraging efficiency

Many measurements of foraging efficiency/advantage rely on an individuals ability to extract energy from a food source. The measure we look at, known as foraging efficiency, is the ratio of energy gained to energy spent and is given mathematically by Laguë et al. [2] as

$$E(t) = \frac{E_{\text{gain}}(t)}{E_{\text{loss}}(t)} = \frac{F(t)}{p_1 t + p_2 t^*}, \tag{5}$$

where $F(t)$ is the total energy gained by foraging for time t, p_1 is the energy cost per unit time during foraging, p_2 is the energy lost per unit time by travel between food patches for time t^*. Note that the marginal value theorem [2, 3] is a simpler version of (5) given by

$$R(t) = \frac{F(t)}{t + t^*}, \tag{6}$$

where $R(t)$ is the rate of energy gain, $F(t)$ is the total energy gained by foraging a patch of food in time t, and t^* is the time to travel between patches.

However, (5) is not spatially explicit, we thus need to convert our spatially explicit

equations and derive a value for individual energy gain. We first do this with a more general case before substituting our equation for consumption, (1c). To begin, let the subscript $\cdot_g$ denote gregarious locusts and $\cdot_s$ denote solitarious. Then, let $f_g(c, s, g)$ be a function describing the energy gain for gregarious locusts per unit area per unit time (the derivation for solitarious is similar and thus omitted here). We can then calculate the total instantaneous gregarious energy gain, I_g at time t as

$$I_g(t) = \int_\Omega f_g(c, s, g) \; d\boldsymbol{x}.$$

This allows us to calculate the average gregarious individual's instantaneous energy gain by dividing $I_g(t)$ by the total number of gregarious locusts,

$$A_g(t) = \frac{\int_\Omega f_g(c, s, g) \; d\boldsymbol{x}}{\phi_g(t)M},$$

where M and ϕ_g are given by (2) and (3) respectively. We can then calculate the average gregarious individuals total energy gain over a timer the time interval, $[0, t]$, by integrating, giving rise to

$$F_g(t) = \int_0^t \frac{\int_\Omega f_g(c, s, g) \; d\boldsymbol{x}}{\phi_g(\tau)M} \; d\tau. \tag{7}$$

By substituting (1c) into (7) and taking into account only the gregarious contribution to ρ we obtain

$$\begin{aligned} F_g(t) &= \int_0^t \frac{\int_\Omega \kappa c g \; d\boldsymbol{x}}{\phi_g M} \; d\tau, \\ &= \int_0^t \kappa \eta_g(\tau) \; d\tau, \end{aligned}$$

This gives the foraging efficiency as

$$E_g(t) = \frac{\int_0^t \kappa \eta_g(\tau) \; d\tau}{p_1 t + p_2 t^*}. \tag{8}$$

Similarly, for solitarious locusts we obtain

$$E_s(t) = \frac{\int_0^t \kappa \eta_s(\tau) \; d\tau}{p_1 t + p_2 t^*}. \tag{9}$$

To compare the two foraging efficiencies, we define the cumulative relative gregarious foraging advantage as

$$B(t) = \frac{E_g}{E_s} = \frac{\int_0^t \eta_g(\tau)\, d\tau}{\int_0^t \eta_s(\tau)\, d\tau}, \tag{10}$$

assuming κ, t^*, p_1 and p_2 are equal for both solitarious and gregarious. If they are unequal we would end up with some scalar multiple of $B(t)$. It should be noted that this is the definition of cumulative relative advantage from Tania et al. [1].

Finally, as the proportion of the population that is gregarious is changing in time $B(t)$ becomes difficult to interpret. We instead assume that $\phi_g(t)$ is constant over the short interval $[t, t + \Delta t]$ for some $\Delta t \ll 1$, we then let $b(t)$ be the cumulative relative advantage over this interval, which we will term the instantaneous relative advantage to be in line with Tania et al. [1]. We find

$$\begin{aligned} b(t) &= \frac{\int_t^{t+\Delta t} \eta_g(\tau)\, d\tau}{\int_t^{t+\Delta t} \eta_s(\tau)\, d\tau}, \\ &\approx \frac{\Delta t \eta_g(t)}{\Delta t \eta_s(t)}, \\ &\approx \frac{\eta_g(t)}{\eta_s(t)}, \end{aligned} \tag{11}$$

which is the measure we use in the main text. We thus find that an instantaneous relative advantage would lead to a cumulative relative advantage for a fixed gregarious mass fraction, and this in turn would imply a foraging advantage to being gregarious.

We note that the results when using (10) instead of (11) give a similar impression. These are shown in Figure 1. In these simulations $\rho_{amb} = 0.95$ and $\kappa = 0.09$, with the symmetric parameter set. The homogeneous food source is labelled $\omega = 100\%$. Similar to the results presented in Figure 7 in the main text, as time increases so too does the cumulative relative foraging advantage of being gregarious. This effect is increased by the mass of food present but is diminished by the size of the food footprint.

References

1. Tania N, Vanderlei B, Heath JP, Edelstein-Keshet L. Role of social interactions in dynamic patterns of resource patches and forager aggregation. Proceedings of

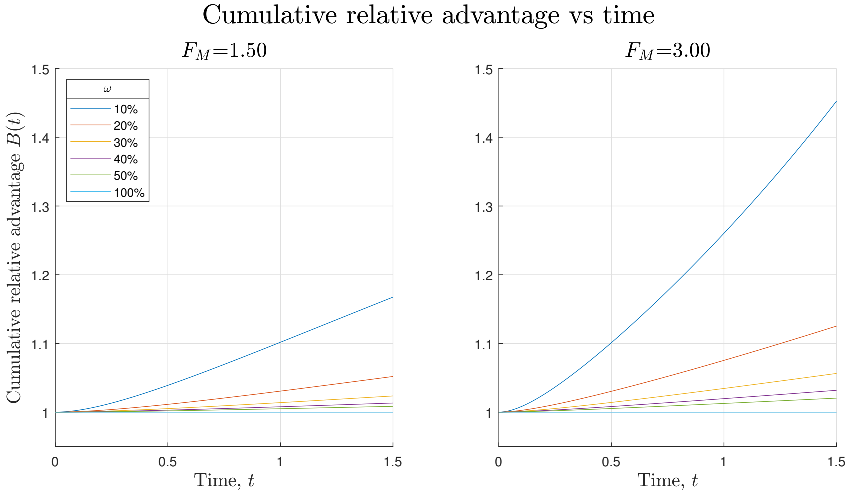

Fig 1. Cumulative relative advantage of gregarious locusts vs time at various food footprints and food masses. In these simulations $\rho_{amb} = 0.95$ and $\kappa = 0.09$, with the symmetric parameter set. The homogeneous food source is labelled $\omega = 100\%$. Similar to the results presented in Figure 7 in the main text, as time increases so too does the cumulative relative foraging advantage of being gregarious. This effect is increased by the mass of food present but is diminished by the size of the food footprint.

the National Academy of Sciences. 2012;109(28):11228–11233. doi:10.1073/pnas.1201739109.

2. Laguë M, Tania N, Heath J, Edelstein-Keshet L. The effects of facilitation and competition on group foraging in patches. Journal of Theoretical Biology. 2012;310:88–96. doi:10.1016/j.jtbi.2012.06.024.

3. Charnov EL. Optimal foraging, the marginal value theorem. Theoretical population biology. 1976;9(2):129–136.
