# Supplementary material for: Modelling locust foraging: How and why food affects group formation
Source: PLoS Comput Biol. 2021 Jul 7;17(7):e1008353. doi: 10.1371/journal.pcbi.1008353 (PMC8289112; doi:10.1371/journal.pcbi.1008353)
Supplement: Attachment — Submitted filename: ResponseLetter.pdf [file pcbi.1008353.s005.pdf]

Dear Prof Couzin,

Thank you for your response regarding our manuscript [PCOMPBIOL-D-20-01682] on the 30/12/2020. Please find attached to this letter a revised manuscript that addresses all your comments and those of the Referees.

We have hopefully addressed both your and reviewer 1's main concern about the validity of the model by better highlighting our assumptions and when the model is most applicable.

Kindest Regards,
Fillipe Georgiou

Editor

That is, one could argue that the approach here has failed to replicate even the basic phenomena seen in real swarms, but this in itself serves the purpose to highlight how and why these major discrepancies may have arisen. Consequently I would strongly recommend the authors reconsider how the paper is structured and should use the locusts more of a source of inspiration, and they should discuss openly such major difference seen between the natural and model systems. This model should not be 'sold' as a model of actual locusts, but rather is inspired by them and acts as a valid and useful exercise in determining how simple rules scale to macroscopic features. This would, in my mind, resolve the issues raised both by reviewer 1 and my own feelings about the manuscript

Thank you for the feedback on the model. We have included many adjustments to the text that hopefully addresses your concerns in this area. In the paper we have used a kinematic model for our non-local locust interactions which fails to capture collective movement dynamics such as alignment. Prior to gregarious aggregation and hopper band formation the model is most applicable as the locusts are in a disordered state and alignment doesn't play a large role in their movement behaviour. Once the hopper band has formed the model doesn't capture the movement dynamics of the swarm but steady state solutions of the model give insights into the behaviour of higher order models which do capture these collective movement dynamics. Specifically, the steady state solutions of the kinematic aggregation equation correspond to the density profile found in the flocking solutions (collective movement of individuals in a single direction), see Carrillo, J. A., S. Martin, and V. Panferov. "A New Interaction Potential for Swarming Models." Physica D: Nonlinear Phenomena, Emergent Behaviour in Multi-particle Systems with Non-local Interactions, 260 (October 1, 2013): 112–26. https://doi.org/10.1016/j.physd.2013.02.004. Section 2.1 flock and mill states.

One further issue that needs to be addressed, and readily can, is the use of "evolution". It should be removed. This work does not consider evolutionary dynamics in an appropriate way, and as noted above this isn't really a viable model of real swarms nor the selection pressures on them. The costs and benefits could be explored in the way presented here, but please note the valid concern of reviewer 1 who notes that you may simply be getting our what you put in. Note also that there has been work on the evolution of phase polyphenism and cannibalism (Guttal et al. (2012) "Cannibalism can drive the evolution of behavioural phase polyphenism in locusts" Ecology Letters) which could be considered when discussing costs and benefits, and previous work that has modelled evolution of locust swarming.

We have removed all talk of evolution, however we have left in the foraging advantage results.

Reviewer 1

The manuscript entitled "Modelling locust foraging: How and why food affects hopper band formation" by Georgiou et al describes analytical and numerical studies of a PDE model for coarse-grained locust swarm dynamics. The model describes the evolution of the density of locusts (either gregarious or solitarious) and food. Analytically, it is shown that, assuming homogeneous and constant densities, the model admits maximal and minimal mass limits. The stability of the homogeneous and constant solutions is also studied. Numerically, the authors solve a 1D version of the model. In particular, the dependence of results on the initial food distribution is studied.

Thank you for the feedback on the model, we have hopefully addressed your concerns about the applicability.

Major Comments

Note	Response notes
I find the paper lacks a well-defined focus or goal. This starts with the abstract, which reads like an introduction. The main results are not explained clearly.	We have re written the abstract to give clearer results.
The figures are difficult to interpret (small font, the captions do not explain what the reader is supposed to look at).	Fixed the figures, increase font size and fontweight for all text.
While the many parameters are defined in the text, the reader should not be expected to remember everything (for example, write the meaning of the parameters in Table 1).	Added variable description reminder text to the table.
The discussion is somewhat scattered and lacks a concrete scientific claim. These comments may be addressed by a through revision.	We have rewritten the discussion to address your concerns
While the mathematics seems of high quality and the biological background is solid and well explained, the connection between the two is loose. Here are a few examples.	
There is no clear definition or explanation what the authors mean by band formation. As far as I understand, they refer to the case in which the fixed, homogeneous solution is unstable. Other studies, e.g. [17] and [33], refer to a moving band as a traveling wave solution with a given density profile. Therefore, the rigorous stability analysis, while mathematically valid, cannot be applied to band formation in realistic locust swarms.	We have added "Similarly, in our work we focus on the formation of aggregation of gregarious locusts, visualised as a clump of gregarious locusts, rather than on the collective movement dynamics in hopper bands." to the introduction. We have hopefully clarified that we are focussing on the formation of hopper bands. The kinematic model that we are using is most applicable prior hopper band formation when locusts are in a more disordered state. Once hopper bands are formed then the model no longer captures movement, however it can give insights into how models that do capture movement will behave.

	The linear stability analysis in a similar fashion to Topaz et. Al (2012) gives the condition for instability of the homogeneous steady state. If the system meets this condition, then there is likely aggregations of gregarious locusts forming.
The numerical simulations are one dimensional. This is perfectly reasonable for academic interests or for modelling marching in circular arenas. However, the relation to realistic moving bands is marginal. In particular, I cannot see how 1D simulations can be used to infer the evolutionary advantages of the specific band width observed in nature.	We no longer make any claims about the evolutionary advantage of gregarisation. We have left the foraging advantage in as it is an interesting result and leaves the door open for future exploration.
Fig. 6 shows situations in which the gregarious and solitarious locusts occupy the epoch of the band (at x=10) simultaneously. While I understand this result mathematically, I am not sure how realistic is. Depending on species, solitarious individuals may be present in dilute areas of a band, e.g. in the rear [17], but not at the most dense parts.	In the case where a hopper band has formed (omega = 10, t= 12.5), solitarious locusts are not present in the hopper band. In the other cases hopper bands failed to form and the gregarious peak is just a nucleus of gregarisation which doesn't lead to a band.<br><br>We have hopefully clarified what we interpret as hopper band formation in the introduction, “Similarly, in our work we focus on the formation of aggregation of gregarious locusts, visualised as a clump of gregarious locusts.”
The conclusions on the benefits of gregariousness for foraging seem to be a direct consequence of some modelling assumptions. In simulations, the initial food is concentrated in a given region (a single patch) and both gregarious and solitarious locusts are attracted to it. Therefore, if gregarious animals are attracted to each other while solitarious animals are repelled, then more gregarious individuals will be around the food. I expect this result will change if several food patches are introduced.	As the locust interactions are based on combined density (s+g), prior to hopper band formation the density of locusts is approximately uniform all the locust interaction terms are small and the locust behaviour is driven by the food interaction term. A similar result is seen with two food patches, see image below (food is green, solitarious locusts are blue, and gregarious locusts are red)<br>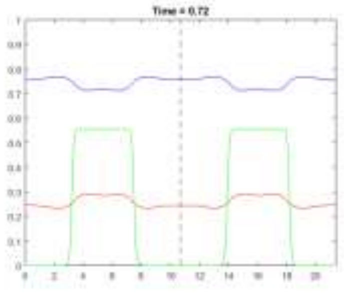

Minor Comments

Note	Response - notes
Eqs. (5) and (6) are the same as (1) and (2).	Deleted Eqs (5) and (6)
Below (6): (3a) is referred to twice.	Fixed
Several assumptions underlying the model should be explained and discussed. Making simplifying assumptions is fine. However, in the very least, it should be pointed out that they may not hold in realistic locust swarms. Here are several examples:	We have included a new assumptions section and added further exploration of the model to our discussion section.
Page 8: I do not see any reason to assume the law of mass action except that it is simple. In particular, the system is not at equilibrium.	We went with the law of mass action as it is the simplest local repulsion term (and akin to a contact repulsion in which both parties avoid each other, rather than a pressure based repulsion) that gives a bounded locust density with increasing mass when used in conjunction with the kinematic model.
Locust phase polyphenism is continuous.	It is and this could be a further exploration of the model, the inclusion of a polyphenism dimension
The model assumptions regarding the effect of food on the movement of locusts should be discussed in light of the following recent highly relevant paper: Dkhili, J., Maeno, K. O., Hassani, L. M. I., Ghaout, S., & Piou, C. (2019). Effects of starvation and vegetation distribution on locust collective motion. Journal of Insect Behavior, 32(3), 207-217.	We have made it clearer that the model is most applicable prior to hopper band formation when the locusts are in a more disordered state. We do mention this as a possible further extension of the model by including hunger based movement.
Page 9, last line: Relate the constants taken from [31] to the proper experiments.	The constants taken from [31] can be considered arbitrary, we do reference them when estimated values are used later in the numerical simulations.
Why is it OK to ignore the diffusion?	For mathematical tractability, while diffusion does still play a role the resulting equations were not solvable with known techniques. We have added “In addition, to be able to solve the resulting equations we ignore the effect of linear diffusion within \(\Omega\), while this gives a less accurate approximation it still shows the effect of food on maximum density.”
Page 12: Explain the meaning of small and large mass limits. It is not clear	Added a definition of total mass and the statement “(i.e. as \(M\rightarrow 0\) and \(M\rightarrow \infty\), respectively)”
The sub-section showing that the center of mass does not move is a nice exercise, but the result is trivial considering the symmetry of the model and initial conditions. The two-page derivation can be put in an appendix.	Changed the sub-section to “Conservation properties” and highlighted that both mass and center of mass are conserved. Derivation left entirely in appendix 2.

Figs 4 and 5: I see only two colors – blue and green. Are there only two states or a continuum of values, as the color-bar suggests?	There is a continuum of values however due to the nature of the gregarious density in the model, the values are contained in the two extremes.

Reviewer 2

This manuscript derives and analyzes a mathematical model (PDE) for locust foraging and interaction that extends the work of Topaz et al. 2012. The model includes both gregarious and solitarious locust densities, a food resource density, and both local and non-local aggregation and repulsion effects. The paper's focus is primarily on mathematical analysis of the model and is quite extensive while staying within the context of those qualitative features that are relevant to the biological system being represented. My feeling is that the manuscript is a fine contribution to the field and should be accepted following a number of mostly minor revisions. The main topics of my comments center around the derivation of the spatial flux terms in the model along with a pervasive need to clarify the language at various points in the paper.

Thank you for your comments which we have hopefully addressed through an edit of the manuscript.

Comments

Notes	Response-Notes
Starting around line 121, there is some general confusion about this term T with the subscript i and the plus/minus superscript (here, I will just refer to it as T). On line 121 it is referred to as a rate. Then on line 124, alpha and beta are introduced and said to be probabilities of movement. This is really vague… movement of what, going where? And how does this result in a rate on the left hand side of the equation? What are the units of this rate? Then on line 131, tau is defined via a law of mass action, but there is no constant of proportionality. Perhaps the authors intend that it will be subsumed into beta, but then beta should not be a probability. Then on line 132, T is called a transition probability. So which is it? A transition probability, or a rate? The distinction matters: T is a nonlinear function of rho. So if we are being asked to consider rho as a stochastic variable (being a sum of the stochastic variables s and g), the expected value of the transition probability T is not equal to the RHS of the equation using the expected value of rho. For a nice explanation on this, see the seminal paper by Mollison (1977), "Spatial contact models for ecological and epidemic spread" in J. R. Statist. Soc. B, particularly the section Relations	Fixed this, T is a transition probability and have included a clarifying brackets "(i.e. the probability that the locust will leave site i)" to give a better idea of what it means.<br><br>In addition we have added "(this should however be only considered an asymptotic approximation)"<br><br>Alpha and beta have been corrected to constants and T to a probability.<br><br>We intended that it will be subsumed into beta and have added "we assume that any constant of proportionality will be subsumed into beta", and beta has been corrected to be a constant.<br><br>We have included the full derivation in a new appendix S1, in general we Taylor expand every term and then take the limit as Delta t, Delta x -> 0 using a parabolic scaling to obtain our expression. This parabolic scaling means that it is possible for an individual to have infinite speed and thus it should only be considered and asymptotic scaling (as per Codling, Edward A and Plank, Michael J and Benhamou, Simon "Random walk models in biology"). We mostly

Between Stochastic and Deterministic Models. So I would ask that the authors please make this section more rigorous. I would also request that there be more detail in the derivation of the continuum limit - this is quite a jump since there is both a spatial and temporal limit occurring, and it is not at all clear how alpha and beta (which I'm already fuzzy on) translate to D and gamma.	consider results around steady states and roughly homogeneous densities, so it is still likely to be a valid approximation.
Equation 14: I am really not sure where this E expression is coming from… it seems to appear a bit out of the blue. Can you provide some more guidance? Also, I would suggest writing E(g) instead of E[g], as with brackets this often means "the expectation of g".	Added a sentence outlining that E is an energy functional (function of a function), it is a technique known as gradient flows. We have included some more references in this section.
Comments relating to language:<br>- Abstract: do you want a colon after "These are" in the third line…?<br>- Line 59: "It is based on"<br>- Equation (5) and (6) and the language surrounding them are a repeat from Equation (1) and (2)<br>- Line 102: Bold J for non-local flux<br>- Line 105: grammar<br>- Line 114: grammar (comma splice)<br>- Line 134: if -> of<br>- Line 162: center of mass<br>- Line 164-166. The meaning of this sentence is not clear to an external reader. What does it mean to find these maximums for "large" and "small" numbers of locusts? This makes sense later on, but the first time you refer to it is here, and it's really confusing.<br>- Line 167: What is the practical effect of assuming c is constant and not depleting? That is, what biological scenario does this correspond to and why can/should we assume this? I'm just not sure why this assumption should be made, and a sentence or two giving context would be helpful.<br>- Line 171: grammar (comma splice)<br>- Line 203-204: wording<br>- Line 208: Same comment as line 167<br>- Line 210: Odd wording given the previous sentence. Please make this paragraph more easy to follow.<br>- Line 215: What is an "upper locust density"? Be a bit more descriptive..?	Abstract: Changed to a colon,<br>59: Added is<br>Removed eq5 and 6<br>102:Bolded J<br>105: NOT fixed yet<br>114: Changed to semicolon<br>134: Changed if to of<br>162: Added the of<br><br>164-166: Added a definition of total mass and the statement "(i.e. as \(M\rightarrow 0\) and \(M\rightarrow \infty\), respectively)"<br><br>167 and 208: To the intro paragraph of the PDE model analysis section we have added "In this section we investigate the behaviour of our model with a spatially uniform and temporally constant food density. This assumption corresponds to environments where the lengthscale of the food footprint is larger than the lengthscale over which the locusts are distributed, and where the timeframe of food consumption is negligible compared to the speed of locust interactions. Aside from simplifying the analysis, this assumption also provides a baseline with which to compare our later results, and hence assess the impact of a patchy food distribution."<br><br>171: changed the comma to "; consequently"<br><br>203-204: changed to "In the simulations we estimate the finite support, \(\Omega\), as the region where \(g > 0.01\)."

- Line 221-222: This was a bit hard to follow in my reading, perhaps because it sounds a little like "when there are a lot of locusts in an area, there can't be a lot of locusts in an area". Maybe just provide some context. Biologically, why should we expect a maximum homogenous locust density under which locust aggregations can form? And make it clear what you mean by "locust aggregations."<br>- Line 257: "we find an alternate expression"<br>- Line 268: comma splice<br>- Fig. 4 caption: why was the final time chosen to be 12.5? This seems like a rather odd number. Also, this wasn't mentioned in the text anywhere… just in the caption?<br>- omega: if it's a percent, write the numerical value with a percent sign. E.g. omega = 0% to 50%. This helps your reader follow what these things are. Same comment for all numerical expressions of omega down to Fig. 7.	210: Rewritten section<br><br>215: Removed from here and moved to 221<br><br>221: Rewitten and added a physical interpretation of this result. "This would likely correspond with an environment so thick with locusts that there isn't room for hopper bands to form."<br><br>257: Fixed<br>268: Split into two sentences.<br><br>Fig 4: the final time was chosen as 50 hours in dimensional terms, this ends up as 12.5 in nondimensional time. As our assumption of locust interactions not being dependant on time, after 50hours our model is not really valid anymore.<br><br>Omega: included % sign, updated plots

Reviewer 3

This paper makes a nice contribution to the locust modeling literature; it is the only agent-based model I know of at the moment that includes attraction/repulsion, resources and phase changes (gregarisation) and starts providing a framework for studying how food distribution can mediate the large-scale gregarisation that mediates the formation of hopper bands.
Perhaps one of the greatest challenges at present in the mathematical modeling of biological population models is developing models that can give robust insight biological processes; the difficulties of identifying appropriate models and parameters from the biology and then sampling the often high-dimensional parameter space should not be underestimated. As such, the fact that this paper leaves me asking for more is a sign of its strength. The conclusion here is that i) resource distribution can mediate the threshold for large-scale gregarisation and ii) there is a foraging advantage for gregarized locusts in certain resource distribution scenarios.

Thank you for your comments which we have hopefully addressed through an edit of the manuscript.

Comments

Notes	Response-Notes
Throughout the paper – the authors should make the spelling of gregarize/gregarise and solitarize/solitarise (and gregarisation/solitarisation etc.) uniform.	Homogenised around the 's' spelling
Abstract: The authors write "It is these short time-scale locust- resource relationships and their effect on hopper band formation that are of interest." Of interest to whom? Passive voice here is confusing/uninformative.	Changed the abstract to remove passive voice and better articulate the results
Line 16: " . . . process of transition called gregarisation." Could the authors add another sentence of explanation here – state clearly what causes gregarisation and perhaps address the timescale on which it occurs, note the process is reversible, and note that solitarisation may take place on a longer timescale. This will foreshadow some of the results investigated later.	Added to the introduction, "Gregarisation is brought about by locusts crowding together and can be reversed by isolating the individuals In the Desert locust, gregarisation can take as little as 4 hours with the timeframe for reversal dependant on the length of time the individual has been gregarious (again, potentially as little as 4 hours)."
Line 48 -51: Do the authors wish to make clear in the introduction that there are many species of locusts and that the observations referred to here are specifically for S. gregaria?	Changed the introduction to include "The model is based on an idealised locust which has both long and short range locust interactions and only interacts with food when it come into direct contact with it."
Line 67-68: There is a potential for confusion here of the meaning of multiple species (mathematical vs. biological) – perhaps multiple component/multiple populations might be better. In the abstract the authors use the language "multiple populations" – why not use that language here also.	Changed to multiple interacting populations

Line 100: For the global mass fraction to make sense you've assumed the total locust mass is finite. Is the domain finite here? Periodic? As you use this quantity later in the stability calculation for an infinite domain of constant mass fraction it might be worth a comment to make the definition consistent.	We have defined the global mass fraction in a slightly different way and then introduce the equivalent definition in the case of homogeneous steady states for the linear stability analysis.
Line 102: Another assumption in this model is that the behaviors are constant in time. If we are considering action over multiple days, that certainly isn't true (for example, locusts certainly feed more and are more active in the day when it is warmer). To be clear, I think from a modelling perspective this is fine, but I believe it should be called out as an assumption.	Excellent point, included this in the new assumptions paragraph at the start of the model derivation section. We also highlight this in the discussion when we suggest further ways to develop the model.
Lines 110-112 appear to be a repeat of lines 99-101.	FIXED-removed second instance
Lines 113 and 114: It says "in Eq (3a) 113 and Eq (3a)" but probably means "in Eq (3a) 113 and Eq (3b)"	FIXED
Line 132: The authors assume that the local behaviors are the same for gregarious and solitarious locusts. While one could argue the diffusive type behavior (which is not social) might be the same (even though gregarious locusts I believe are more active) the second term (which is related to collisions) arguably should be different for the two behavioral phases. I'm all in favor of simplifying assumptions, but the authors might address this point.	Again, we have included this in the new assumptions paragraph at the start of the model derivation section. We also highlight this in the discussion when we suggest further ways to develop the model.
Equations 8a & 8b: I am reasonably sure that there is an extra divergence operator in this expression – shouldn't they be vectors (and therefore multiples of the gradients in the spatial variable)?	FIXED-Removed second divergence operator
Lines 134-141: I'm a little confused here – the authors have in essence included both local and non-local repulsion. Repulsion is sometimes thought of biologically as collision avoidance (which is the essence of the local derivation here). Perhaps the authors could clarify why they include both effects?	We include both as there is a non-local aspect of avoidance (as per the Buhl paper) however the non-local operator doesn't capture this behaviour (as Nabla Q(0)=0). This could also be interpreted as the visual and contact aspects of repulsion
Line 246-247: I suspect the statement about boundary conditions and center of mass here is incorrect – consider just the diffusion equation in 1D. You need to vanish – integrating by parts twice yields boundary terms like x\rho_x - \rho suggesting the correct statement is that you need both \rho and \rho_x to vanish at the	Sorry the proof only works on an infinite domain with \rho->0 at infinity and a bounded mass, we have corrected this and relegated it to the S2 appendix as per reviewer 1's request.

boundary of the domain. Moreover, if the diffusion constant (D) here is non-zero the support of the solution nearly certainly becomes the entire domain after an infinitesimal amount of time. So the statement here is probably only correct if: 1) D=0 and the solution is compactly supported or 2) D>0, the domain is infinite and the mass is finite (an analyst might want a bound on the density gradient also but an applied mathematician should be happy).	
Line 284: Returning to the question of the two repulsive behaviors it might be interesting to investigate the relative sizes with these parameters of the effects of diffusion, collision avoidance (local non-linear repulsion) and non-local repulsion in the stability criteria below (225) and in numerical studies. This referee realizes this may be a big “ask” – however if the authors have any insight on this issue, I’d encourage them to include it.	We haven’t had the time to perform a parameter sensitivity analysis. We do however have some preliminary results where we used the unchanged main parameters from the Topaz paper, this corresponds to an increase in local and non-local repulsion what we saw over the range tested was a narrower but taller optimal width. In other experiments, lowering the local repulsion would lower the minimum density required but not shift the location of the peak.
Similarly, anything the authors can say about parameter sensitivity of these results would be of interest. I understand the computational/human cost may be prohibitive.	We haven’t done a parameter sensitivity analysis but understanding what parameters the greatest effect on this optimal width have would be an excellent avenue of further exploration of the model.
In Figures 4 & 5 I am a little concerned about the difference between “Maximum gregarious locust density” and “Final peak density” – I believe the authors just took the maximum at the final time step of the simulation. Can you convince the reader this is the right measure? How do I know that the swarm didn’t gregarize and then somehow dissipate?	Changed the plots to be maximum peak gregarious density over the whole batch of simulations, value wise they remain the same as before.
Similarly, in Figures 4 & 5 and looking at the third figure in the fourth row of Figure 6, can the authors predict the peak density from the analysis in Figure 3? How do they compare?	The mass of locusts in figure 6 sits in the area where neither approximation is particularly effective.
Line 322: The authors state: “as we found in certain cases the initial noise had an effect on whether a hopper band would form.” – yet Figures 4 & 5 only report one run (I believe). How did you report data in cases where sometimes you have a hopper band form and sometimes not?	Added the following text in the numerical section “In each simulation, the solitarious and gregarious populations very quickly tend to an almost smooth and symmetric distribution around the food, however a small quantity of noise persists across the population and this breaks the symmetry. As we found in certain cases the initial noise had an effect on whether a hopper band would form we ran three simulations for each combination of \(\rho_{\text{amb}},\omega,\) and \(F_M\)

	with varied initial noise and took the maximum peak density across the three simulations.”
Figure 6: It seems likely that the symmetry in the problem plays a role here – can the authors comment on the mechanism behind the symmetry breaking seen in the second figure of row 3 and the third figure of row 4?	With the noisy initial conditions a small amount of noise persists across the simulation, so once the instability condition is reached this noise triggers the hopper band formation.
Line 364: Can you reconcile the statement: “This effect is visualised in Fig 6, as gregarious locusts aggregate in the center of the food mass and displace their solitarious counterparts” with the third figure of row 4 where it appears the food source is entirely in the solitary portion of the swarm?	Added a “prior to hopper band formation” as we were a bit ambiguous in what we were referring to.
Line 374: “Analytical investigations of our model shows that a spatially uniform food source has a variety of effects has on locust behavior” – eliminate the second appearance of “has” perhaps?	Corrected
Line 383: “Using this relationship we then found that our model also has a theoretical **maximum** locust density for hopper band formation, and that the presence of food lowers both the required time and density of locusts for hopper band formation.” Should maximum be minimum here? If not . . . you lost me. Please explain.	Has both a maximum and minimum, we have clarified this in the linear stability analysis section and given an interpretation of what this may mean. “This would likely correspond with an environment so thick with locusts that there is insufficient room for hopper bands to form.”
