# Supplementary material for: Modelling locust foraging: How and why food affects group formation
Source: PLoS Comput Biol. 2021 Jul 7;17(7):e1008353. doi: 10.1371/journal.pcbi.1008353 (PMC8289112; doi:10.1371/journal.pcbi.1008353)
Supplement: Attachment — Submitted filename: ResponseLetter.pdf [file pcbi.1008353.s006.pdf]

Dear Jason Papin and Feilim Mac Gabhann,

Thank you for your response regarding our manuscript [PCOMPBIOL-D-20-01682] on the 29/04/2021. Please find attached to this letter a revised manuscript that addresses all the comments of the Referee.

We have hopefully addressed the main concerns about the use of the term hopper band and the measure of foraging efficiency. The editorial process has been very insightful and created a better paper because of it.

Kindest Regards,
Fillipe Georgiou

Reviewer 1:

Reviewer #1: Second report on "Modelling locust foraging: How and why food affects hopper band formation". The manuscript has been improved considerably. I recommend it is accepted to PLoS Comput Biol. I have two comments related to the presentation of the main conclusions.

Thank you for the second review, we have hopefully addressed your concerns in this round of revisions.

1. As the authors explained in their reply letter, the model, which does not consider alignment, is not directly relevant to band formation. Instead, the model addresses aggregation, which is a required step before band formation. For this reason, I find that the repeated reference to band formation, including in the title, is confusing.

While the model is most valid up until the onset of collective behaviour, we have changed all references to hopper bands to gregarious groups (or aggregations). We use the new title 'Modelling locust foraging: How and why food affects group formation'. In addition, we highlight the results that might be applicable to higher order models and hopper bands (specifically, the small and large mass limits).

2. The interpretation of foraging as the "per capita contact with food" within a single food patch is not the standard definition of foraging efficiency. As far as I know, efficient foraging typically refers to the probability or rate of finding a patch. For example, from Wikipedia: "The marginal value theorem (MVT) is an optimality model that usually describes the behavior of an optimally foraging individual in a system where resources (often food) are located in discrete patches separated by areas with no resources. Due to the resource-free space, animals must spend time traveling between patches." Therefore, the authors claim that "there exists a foraging advantage to being gregarious" is misleading.

While we use the measure "per capita contact with food " from Tania et. al. we have attached an appendix (appendix S4) that shows how the measure can be derived from the marginal value theorem (as well as from another more general measure of foraging efficiency).

To give a brief overview of the derivation. The marginal value theorem relies on an individual's ability to extract energy from a food patch. We show that by taking our equations and finding the average food consumption by both gregarious and solitarious locusts. Then comparing the resulting

foraging efficiencies, we can derive the previously used measures. While it does rely on our choice of consumption term, within the context of our paper the results are valid.

Overall, my impression is that the main results are misrepresented.

We have hopefully clarified our results and the methods used to arrive at them.
