# Supplementary material for: Correction: Modelling locust foraging: How and why food affects group formation
Source: PLoS Comput Biol. 2021 Dec 20;17(12):e1009695. doi: 10.1371/journal.pcbi.1009695 (PMC8687532; doi:10.1371/journal.pcbi.1009695)
Supplement: S1 Appendix — The full detailed derivation of the flux terms based on local interactions given in the model derivation section. (PDF) [file pcbi.1009695.s001.pdf]

S1 Appendix: Detailed derivation of local flux

Fillipe Georgiou[1*], Camille Buhl[2], J.E.F. Green[3], Bishnu Lamichhane[1], Ngamta Thamwattana[1],

1 School of Mathematical and Physical Sciences, University of Newcastle, Callaghan, Australia

2 School of Agriculture, Food and Wine, University of Adelaide, Adelaide, Australia

3 School of Mathematical Sciences, University of Adelaide, Adelaide, Australia

* fillipe.georgiou@uon.edu.au

In this appendix we provide the full details of the derivation of the local interaction terms. These are captured by taking the continuum limit of a lattice model, we do this by following the work of Painter and Sherratt [1]. We begin by considering solitarious locust movement on a one-dimensional lattice (we assume that local gregarious locust behaviour is the same resulting in a similar derivation). Let s_i^t be the number of solitarious locusts at site i at time t, and let g_i^t,ρ_i^t, and c_i^t be similarly defined.

We assume that the transition probabilities for a locust at the ith site depends on the food density at that site, and the relative population density between the current site and neighbouring sites. If we let $\mathcal{T}_i^{\pm}$ be the probability at which locusts at site i move to the right, $+$, and left, $-$, during a timestep, then our transition probabilities are

$$\mathcal{T}_i^{\pm} = F(c_i)(\alpha + \beta(\tau(\rho_i) - \tau(\rho_{i\pm 1}))), \tag{1}$$

where F is a function of food density, τ is a function related to the local locust density, and α and β are constants. Then the number of individuals in cell i at time $t + \Delta t$ is given by

$$s_i^{t+\Delta t} = s_i^t + \mathcal{T}_{i+1}^{-} s_{i+1}^t + \mathcal{T}_{i-1}^{+} s_{i-1}^t - (\mathcal{T}_i^{-} + \mathcal{T}_i^{+}) s_i^t. \tag{2}$$

Substituting (1) into (2) gives

$$
\begin{aligned}
s_i^{t+\Delta t} =& s_i^t + F(c_{i+1})(\alpha + \beta(\tau(\rho_{i+1}) - \tau(\rho_i)))s_{i+1}^t \\
&+ F(c_{i-1})(\alpha + \beta(\tau(\rho_{i-1}) - \tau(\rho_i)))s_{i-1}^t \\
&- [F(c_i)(\alpha + \beta(\tau(\rho_i) - \tau(\rho_{i-1}))) + F(c_i)(\alpha + \beta(\tau(\rho_i) - \tau(\rho_{i+1})))]s_i^t. \qquad (3)
\end{aligned}
$$

We the rearrange (3) to take out the common factors α and β, giving

$$
\begin{aligned}
s_i^{t+\Delta t} =& s_i^t + \alpha[F(c_{i+1})s_{i+1}^t + F(c_{i-1})s_{i-1}^t - 2F(c_i)s_i^t] \\
&+ \beta \left[F(c_{i+1})s_{i+1}^t(\tau(\rho_{i+1}) - \tau(\rho_i)) \right. \\
&+ F(c_{i-1})s_{i-1}^t(\tau(\rho_{i-1}) - \tau(\rho_i)) \\
&\left. - F(c_i)s_i^t(2\tau(\rho_i) - \tau(\rho_{i-1}) - \tau(\rho_{i+1}))\right]. \qquad (4)
\end{aligned}
$$

We then Taylor expand the terms in (4) to obtain the equation in relation to the site i at time t only. Beginning with,

$$
s_i^{t+\Delta t} = s_i^t + \Delta t \frac{\partial s_i^t}{\partial t} + \mathcal{O}(\Delta t^2). \qquad (5)
$$

Then for the terms related to α we get

$$
\begin{aligned}
\alpha[\cdot] =& \alpha \left[F(c_i)s_i^t + \Delta x \frac{\partial}{\partial x}(F(c_i)s_i^t) + \frac{\Delta x^2}{2}\frac{\partial^2}{\partial x^2}(F(c_i)s_i^t) + \frac{\Delta x^3}{6}\frac{\partial^3}{\partial x^3}(F(c_i)s_i^t) \right. \\
& F(c_i)s_i^t - \Delta x \frac{\partial}{\partial x}(F(c_i)s_i^t) + \frac{\Delta x^2}{2}\frac{\partial^2}{\partial x^2}(F(c_i)s_i^t) - \frac{\Delta x^3}{6}\frac{\partial^3}{\partial x^3}(F(c_i)s_i^t) \\
& \left. -2F(c_i)s_i^t + \mathcal{O}(\Delta x^4)\right], \\
=& \alpha \Delta x^2 \frac{\partial^2}{\partial x^2}(F(c_i)s_i^t) + \mathcal{O}(\Delta x^4), \qquad (6)
\end{aligned}
$$

as the $0^{th}, 1^{st},$ and $, 3^{rd}$ order terms of Δx cancel each other out. We then turn our attention to our terms involving β, we will Taylor expand each multiplication

individually as otherwise the terms become unmanageable. To begin,

$$
\begin{aligned}
\mathcal{R} =& F(c_{i+1})s_{i+1}^t(\tau(\rho_{i+1}) - \tau(\rho_i)) \\
=& \left[F(c_i)s_i^t + \Delta x \frac{\partial}{\partial x}(F(c_i)s_i^t) + \frac{\Delta x^2}{2}\frac{\partial^2}{\partial x^2}(F(c_i)s_i^t) + \frac{\Delta x^3}{6}\frac{\partial^3}{\partial x^3}(F(c_i)s_i^t)\right] \\
& \cdot \left[\tau(\rho_i) - \tau(\rho_i) + \Delta x \frac{\partial}{\partial x}(\tau(\rho_i)) + \frac{\Delta x^2}{2}\frac{\partial^2}{\partial x^2}(\tau(\rho_i)) + \frac{\Delta x^3}{6}\frac{\partial^3}{\partial x^3}(\tau(\rho_i))\right] + \mathcal{O}(\Delta x^4) \\
=& F(c_i)s_i^t \left[\Delta x \frac{\partial}{\partial x}\left(\tau(\rho_i)\right) + \frac{\Delta x^2}{2}\frac{\partial^2}{\partial x^2}\left(\tau(\rho_i)\right) + \frac{\Delta x^3}{6}\frac{\partial^3}{\partial x^3}\left(\tau(\rho_i)\right)\right] \\
& + \Delta x \frac{\partial}{\partial x}(F(c_i)s_i^t) \left[\Delta x \frac{\partial}{\partial x}(\tau(\rho_i)) + \frac{\Delta x^2}{2}\frac{\partial^2}{\partial x^2}(\tau(\rho_i))\right] \\
& + \frac{\Delta x^2}{2}\frac{\partial^2}{\partial x^2}\left(F(c_i)s_i^t\right)\left[\Delta x \frac{\partial}{\partial x}(\tau(\rho_i))\right] + \mathcal{O}(\Delta x^4),
\end{aligned} \tag{7}
$$

and

$$
\begin{aligned}
\mathcal{L} =& F(c_{i-1})s_{i-1}^t(\tau(\rho_{i-1}) - \tau(\rho_i)) \\
=& \left[F(c_i)s_i^t - \Delta x \frac{\partial}{\partial x}(F(c_i)s_i^t) + \frac{\Delta x^2}{2}\frac{\partial^2}{\partial x^2}(F(c_i)s_i^t) - \frac{\Delta x^3}{6}\frac{\partial^3}{\partial x^3}(F(c_i)s_i^t)\right] \\
& \cdot \left[\tau(\rho_i) - \tau(\rho_i) - \Delta x \frac{\partial}{\partial x}(\tau(\rho_i)) + \frac{\Delta x^2}{2}\frac{\partial^2}{\partial x^2}(\tau(\rho_i)) - \frac{\Delta x^3}{6}\frac{\partial^3}{\partial x^3}(\tau(\rho_i))\right] + \mathcal{O}(\Delta x^4) \\
=& F(c_i)s_i^t \left[-\Delta x \frac{\partial}{\partial x}\left(\tau(\rho_i)\right) + \frac{\Delta x^2}{2}\frac{\partial^2}{\partial x^2}\left(\tau(\rho_i)\right) - \frac{\Delta x^3}{6}\frac{\partial^3}{\partial x^3}\left(\tau(\rho_i)\right)\right] \\
& - \Delta x \frac{\partial}{\partial x}(F(c_i)s_i^t) \left[-\Delta x \frac{\partial}{\partial x}(\tau(\rho_i)) + \frac{\Delta x^2}{2}\frac{\partial^2}{\partial x^2}(\tau(\rho_i))\right] \\
& + \frac{\Delta x^2}{2}\frac{\partial^2}{\partial x^2}\left(F(c_i)s_i^t\right)\left[-\Delta x \frac{\partial}{\partial x}(\tau(\rho_i))\right] + \mathcal{O}(\Delta x^4),
\end{aligned} \tag{8}
$$

and finally,

$$
\begin{aligned}
\mathcal{C} =& -F(c_i)s_i^t(2\tau(\rho_i) - \tau(\rho_{i-1}) - \tau(\rho_{i+1})) \\
=& -F(c_i)s_i^t \Big[2\tau(\rho_i) - \tau(\rho_i) + \Delta x \frac{\partial}{\partial x}(\tau(\rho_i)) - \frac{\Delta x^2}{2}\frac{\partial^2}{\partial x^2}(\tau(\rho_i)) + \frac{\Delta x^3}{6}\frac{\partial^3}{\partial x^3}(\tau(\rho_i)) \\
& -\tau(\rho_i) - \Delta x \frac{\partial}{\partial x}(\tau(\rho_i)) - \frac{\Delta x^2}{2}\frac{\partial^2}{\partial x^2}(\tau(\rho_i)) - \frac{\Delta x^3}{6}\frac{\partial^3}{\partial x^3}(\tau(\rho_i))\Big] + \mathcal{O}(\Delta x^4), \\
=& \Delta x F(c_i)s_i^t \frac{\partial^2}{\partial x^2}(\tau(\rho_i)) + \mathcal{O}(\Delta x^4).
\end{aligned} \tag{9}
$$

Adding (7), (8), and (9), gives

$$\mathcal{L}+\mathcal{C}+\mathcal{R} = 2\Delta x^2 \left[\Delta x F(c_i) s_i^t \frac{\partial^2}{\partial x^2}(\tau(\rho_i)) + \frac{\partial}{\partial x}(F(c_i)s_i^t)\frac{\partial}{\partial x}\left(\tau(\rho_i)\right)\right] + \mathcal{O}(\Delta x^4),$$
$$= 2\Delta x^2 \frac{\partial}{\partial x}\left(F(c_i)s_i^t \frac{\partial}{\partial x}\left(\tau(\rho_i)\right)\right) + \mathcal{O}(\Delta x^4). \quad (10)$$

Combining (5), (6) and 10 into (4), gives,

$$s_i^t + \Delta t \frac{\partial s_i^t}{\partial t} + \mathcal{O}(\Delta t^2) = s_i^t + \alpha \Delta x^2 \frac{\partial^2}{\partial x^2}(F(c_i)s_i^t) + 2\beta \Delta x^2 \frac{\partial}{\partial x}\left(F(c_i)s_i^t \frac{\partial}{\partial x}\left(\tau(\rho_i)\right)\right) + \mathcal{O}(\Delta x^4),$$

which we rearranging to obtain

$$\frac{\partial s_i^t}{\partial t} = \alpha \frac{\Delta x^2}{\Delta t}\frac{\partial^2}{\partial x^2}(F(c_i)s_i^t) + 2\beta \frac{\Delta x^2}{\Delta t}\frac{\partial}{\partial x}\left(F(c_i)s_i^t \frac{\partial}{\partial x}\left(\tau(\rho_i)\right)\right) + \mathcal{O}(\Delta x^4) + \mathcal{O}(\Delta t^2). \quad (11)$$

We then substitute our functions,

$$F(c_i) = e^{-\frac{c_i}{c_0}}, \text{ and } \tau(\rho_i) = \rho_i^2$$

to obtain

$$\frac{\partial s_i^t}{\partial t} = \alpha \frac{\Delta x^2}{\Delta t}\frac{\partial^2}{\partial x^2}\left(e^{-\frac{c_i}{c_0}} s_i^t\right) + 2\beta \frac{\Delta x^2}{\Delta t}\frac{\partial}{\partial x}\left(e^{-\frac{c_i}{c_0}} s_i^t \frac{\partial}{\partial x}\left(\rho_i^2\right)\right) + \mathcal{O}(\Delta x^4) + \mathcal{O}(\Delta t^2). \quad (12)$$

We then take the limit as $\Delta x, \Delta t \to 0$ such that,

$$\lim_{\substack{\Delta x \to 0 \\ \Delta t \to 0}} \alpha \frac{\Delta x^2}{\Delta t} = D, \text{ and } \lim_{\substack{\Delta x \to 0 \\ \Delta t \to 0}} 2\beta \frac{\Delta x^2}{\Delta t} = D\gamma,$$

to find,

$$\frac{\partial s}{\partial t} = D\frac{\partial^2}{\partial x^2}(e^{-\frac{c}{c_0}} s) + D\gamma \frac{\partial}{\partial x}\left(e^{-\frac{c}{c_0}} s \frac{\partial}{\partial x}\left(\rho^2\right)\right). \quad (13)$$

Which we then rearrange to find our flux as

$$J_{s_{\text{local}}} = -D\left[\frac{\partial}{\partial x}\left(s e^{-\frac{c}{c_0}}\right) + \gamma s \rho e^{-\frac{c}{c_0}} \frac{\partial \rho}{\partial x}\right]. \quad (14)$$

The derivation of $J_{g_{\text{local}}}$ follows the same method.

References

1. Kevin J. Painter and Jonathan A. Sherratt. Modelling the movement of interacting cell populations. *Journal of Theoretical Biology*, 225(3):327–339, Dec 2003.
