# Supplementary material for: Correction: Modelling locust foraging: How and why food affects group formation
Source: PLoS Comput Biol. 2021 Dec 20;17(12):e1009695. doi: 10.1371/journal.pcbi.1009695 (PMC8687532; doi:10.1371/journal.pcbi.1009695)
Supplement: S3 Appendix — The full detailed derivation of the numerical scheme used for simulating the numerical results. (PDF) [file pcbi.1009695.s002.pdf]

S3 Appendix: Numerical scheme

Fillipe Georgiou[1*], Camille Buhl[2], J.E.F. Green[3], Bishnu Lamichhane[1], Ngamta Thamwattana[1],

1 School of Mathematical and Physical Sciences, University of Newcastle, Callaghan, Australia

2 School of Agriculture, Food and Wine, University of Adelaide, Adelaide, Australia

3 School of Mathematical Sciences, University of Adelaide, Adelaide, Australia

* fillipe.georgiou@uon.edu.au

In this appendix we provide a detailed derivation of the numerical scheme used. To begin our non-dimensionalised system of equations was given by

$$\frac{\partial g}{\partial t} + \nabla \cdot (g\boldsymbol{v}_g) - D\nabla \cdot \left[e^{-c}\nabla g\right] = -f_1(\rho)g + f_2(\rho)s, \tag{1a}$$

$$\frac{\partial s}{\partial t} + \nabla \cdot (s\boldsymbol{v}_s) - D\nabla \cdot \left[De^{-c}\nabla s\right] = f_1(\rho)g - f_2(\rho)s, \tag{1b}$$

$$\frac{\partial c}{\partial t} = -\kappa c(\boldsymbol{x},t)\rho(\boldsymbol{x},t), \tag{1c}$$

with 1

$$\boldsymbol{v}_g = -\nabla(Q_g \star \rho) + De^{-c}\left(\nabla c - \gamma\rho\nabla\rho\right),$$

and 2

$$\boldsymbol{v}_s = -\nabla(Q_s \star \rho) + De^{-c}\left(\nabla c - \gamma\rho\nabla\rho\right),$$

with our specific functions given by

$$Q_g = R_g e^{\frac{-|\boldsymbol{x}|}{r_g}} - A_g e^{-|\boldsymbol{x}|}, Q_s = R_s e^{\frac{-|\boldsymbol{x}|}{r_s}},$$

$$f_1(\rho) = \frac{\delta^*}{1+\rho^2}, f_2(\rho) = \frac{(\rho k)^2}{1+(\rho k)^2}.$$

1 Numerical Scheme

We now derive the numerical scheme for (1a) in one dimension using a finite volume method (FVM). The numerical scheme for (1b) is similar and thus omitted here. For the numerical scheme the terms described in Table 1 are used in relation to an arbitrary cell i with cell boundaries $i \pm \frac{1}{2}$.

Symbol	Definition
Δx	spatial size of cells in the x direction
$\mathbf{x}$	vector representing the discretised spatial grid
x_i	x value of the midpoint of a grid cell
S_i, G_i, C_i	Approximate function values of s, g, and c
$\mathbf{S}, \mathbf{G}$	vectors representing the discretised functions s and g
$\mathscr{L}_i$	Approximate value of the local component of the $\mathbf{v}_g$
$\mathscr{N}_i$	Approximate value of the non-local component of $\mathbf{v}_g$
$\mathscr{A}_i$	Approximate value of the advective component of the equation
$\mathscr{D}_i$	Approximate value of the diffusive component of the equation
$\mathscr{K}_i$	Approximate value of the kinetic component of the equation

Table 1. Definitions of symbols used in numerical scheme at arbitrary cell i.

Beginning with the local part of the velocity term (denoted $\mathscr{L}$)

$$\mathscr{L} = De^{-c}\left(\frac{\partial c}{\partial x} - \gamma\rho\frac{\partial \rho}{\partial x}\right),$$

we approximate both derivatives using central differencing schemes, giving

$$\mathscr{L}_i \approx De^{-C_i}\left(\frac{C_{i+1} - C_{i-1}}{2\Delta x} - \gamma(S_i + G_i)\frac{S_{i+1} + G_{i+1} - S_{i-1} - G_{i-1}}{2\Delta x}\right),$$

at an arbitrary cell i. Then, for the non-local component of the velocity term (denoted $\mathscr{N}$),

$$\mathscr{N} = -\nabla(Q_g \star \rho),$$

we begin by exploiting the convolution theorem, which states that under suitable conditions the Fourier transform of a convolution of two functions is equal to the point-wise product of their individual Fourier transforms, i.e.,

$$\mathscr{F}\{f * g\} = \mathscr{F}\{f\} \cdot \mathscr{F}\{g\},$$

where $\mathscr{F}$ represents the Fourier transform (we also denote the inverse Fourier transform

as $\mathscr{F}^{-1}$). Additionally, we use the following property of convolutions

$$\frac{\partial}{\partial x}(f * g) = \left(\left(\frac{\partial}{\partial x} f \right) * g \right) = \left(f * \left(\frac{\partial}{\partial x} g \right) \right),$$

to turn the convolution component of the advection term into

$$\mathscr{N} = \mathscr{F}^{-1} \left\{ \mathscr{F} \left\{ -\frac{\partial}{\partial x} Q_g \right\} \cdot \mathscr{F} \{\rho\} \right\}.$$

We can then approximate the convolution as

$$\mathscr{N} \approx \text{real} \left\{ \text{ifft} \left\{ \text{DFT} \left\{ -\frac{\partial}{\partial x} Q_g(\mathbf{x}) \right\} \cdot \text{fft} \{ \mathbf{S} + \mathbf{G} \} \right\} \right\},$$

where fft and ifft represent the fast Fourier transform and inverse fast Fourier transform respectively, and DFT represents the discrete Fourier transform. We take only the real component of the ifft as any imaginary value will simply be due to error. We also require $Q_g(x)$ to be periodic on the domain $x \in [0, L]$. We use the discrete Fourier transform (DFT) derived by [1]:

$$\text{DFT} \left\{ -\frac{\partial}{\partial x} e^{-\frac{|x|}{r}} \right\} = -\frac{i \Delta x \sin(\Delta x q)}{r \left(\cosh(\Delta x / r) - \cos(\Delta x q) \right)},$$

where q is our frequency domain. By combining the local and non-local components and letting

$$F_i = (\mathscr{L}_i + \mathscr{N}_i) G_i$$

we can approximate the wavespeed at a cell boundary, $i - \frac{1}{2}$, as

$$P_{i-\frac{1}{2}} = \frac{F_i - F_{i-1}}{G_i - G_{i-1}},$$

and the wave size as

$$W_{i-\frac{1}{2}} = G_i - G_{i-1}.$$

From this we can approximate the advection component using an upwinding scheme, giving

$$\mathscr{A}_i = \frac{1}{\Delta x} \left(\max\{P_{i-\frac{1}{2}}, 0\} W_{i-\frac{1}{2}} + \min\{P_{i+\frac{1}{2}}, 0\} W_{i+\frac{1}{2}} \right)$$

Next, for the diffusion term, $\mathscr{D}$

$$\mathscr{D} = D \frac{\partial}{\partial x} \left[e^{-c} \frac{\partial g}{\partial x} \right]$$

We can approximate this using FVM as

$$\mathscr{D}_i \approx \frac{D}{\Delta x^2} \left(e^{-\frac{C_{i-1}+C_i}{2}} (G_i - G_{i-1}) - e^{-\frac{C_{i+1}+C_i}{2}} (G_{i+1} - G_i) \right)$$

Then the kinetic component is given by

$$\mathscr{K}_i = -f_1(S_i + G_i)G_i + f_2(S_i + G_i)S_i$$

Combining all the terms we obtain,

$$G_i^{t+\Delta t} = G_i^t - \Delta t(\mathscr{A}_i + \mathscr{D}_i - \mathscr{K}_i)$$

Finally, for we use an adaptive Dormand-Prince method [2] for the time component.

References

1. Chad M. Topaz, Maria R. D'Orsogna, Leah Edelstein-Keshet, and Andrew J. Bernoff. Locust dynamics: Behavioral phase change and swarming. *PLOS Computational Biology*, 8(8):e1002642, Aug 2012.

2. Dormand, J. R. and P. J. Prince (1980, Mar). A family of embedded runge-kutta formulae. *Journal of Computational and Applied Mathematics 6*(1), 19–26.
